# Cyclic AMP response element-binding protein (CREB) transcription factor in astrocytic synaptic communication

**DOI:** 10.3389/fnsyn.2022.1059918

**Published:** 2023-01-04

**Authors:** Jooyoung Kim, Bong-Kiun Kaang

**Affiliations:** School of Biological Sciences, College of Natural Sciences, Seoul National University, Seoul, South Korea

**Keywords:** astrocyte, CREB, tripartite synapse, GPCR, PIP_3_ signaling

## Abstract

Astrocytes are known to actively participate in synaptic communication by forming structures called tripartite synapses. These synapses consist of presynaptic axon terminals, postsynaptic dendritic spines, and astrocytic processes where astrocytes release and receive transmitters. Although the transcription factor cyclic AMP response element (CRE)-binding protein (CREB) has been actively studied as an important factor for mediating synaptic activity-induced responses in neurons, its role in astrocytes is relatively unknown. Synaptic signals are known to activate various downstream pathways in astrocytes, which can activate the CREB transcription factor. Therefore, there is a need to summarize studies on astrocytic intracellular pathways that are induced by synaptic communication resulting in activation of the CREB pathway. In this review, we discuss the various neurotransmitter receptors and intracellular pathways that can induce CREB activation and CREB-induced gene regulation in astrocytes.

## Introduction

Astrocytes are glial cells of the brain that perform diverse functions, including supportive functions such as brain barrier transport, energy molecule transport, ion concentration control, and neurotransmitter recycling. In the last two decades, the role of astrocytes, in addition to their supportive functions, has been largely studied. Molecular and behavioral studies have shown that astrocytes are major participants in information processing and learning in the brain. The astrocytic process establishes a structure morphologically and functionally similar to neuronal synapses, called tripartite synapses, which bidirectionally affect synapses using release factors and contact-dependent factors. Through tripartite synapses, astrocytes respond to neuronal activity and various neurotransmitters. A single astrocyte can occupy up to 100,000 synapses, suggesting that astrocytes may be the center for converging various neuronal inputs and modulating synapses (Bushong et al., 2002). Many studies have shown that neighboring neuronal activity can elicit calcium activity in astrocytes, which is a major signaling mechanism (Martin et al., 2015; Martin-Fernandez et al., 2017; Lezmy et al., 2021). Astrocytic calcium signals mediate differential gene expression, structural changes, and release of signal molecules called gliotransmitters.

**FIGURE 1:**
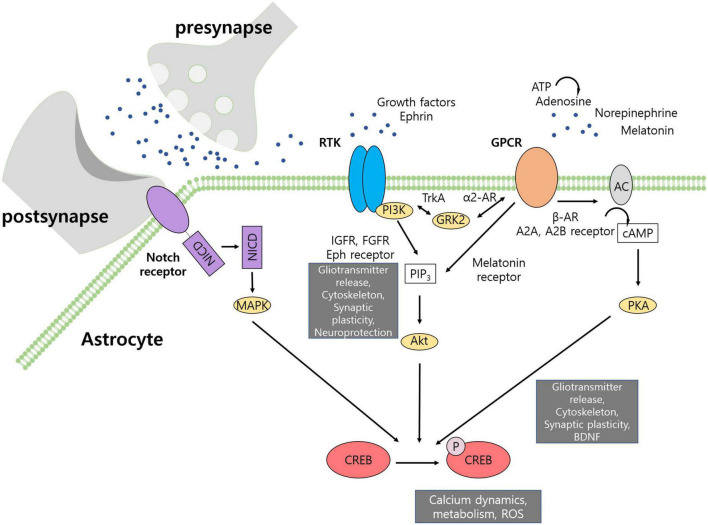
Activation of CREB pathway in astrocyte. CREB activity regulates gliotransmitter release, cytoskeleton dynamics, synaptic plasticity, neuroprotection, growth factor release, calcium dynamics, metabolism, and generation of reactive oxygen species. Receptor tyrosine kinases produce PIP_3_ to activate Akt that can phosphorylate CREB. GPCRs can activate adenylyl cyclase to produce cAMP and activate PKA. Some GPCRs, such as melatonin receptor, can activate CREB *via* Akt. Notch intracellular domain is cleaved upon activation by contact-dependent signals and may activate MAPK pathway.

The CREB transcription factor, known to be involved in neuronal plasticity, is also activated in astrocytes in response to neurotransmitters (Carriba et al., 2012). CREB binds to CRE of the promoter to regulate transcription, and is activated by phosphorylation at Ser-133 (Gonzalez and Montminy, 1989). Cyclic adenosine monophosphate (cAMP)/protein kinase A (PKA) is known to activate CREB *via* the cAMP/PKA pathway; however, other factors such as phosphatidylinositol 3-kinase (PI3K)/Akt can also phosphorylate CREB (Figure 1). In neurons, apart from activating neuronal plasticity genes, including BDNF, overexpression of CREB has been shown to be sufficient to allocate memory-encoding cells (Han et al., 2009). Although neuronal CREB has been actively studied, in astrocytes, the CREB transcription factor has rarely been reported in relation to synaptic communication and plasticity. Genes related to amino acid processing, cytoskeleton dynamics, and vesicle dynamics have been shown to be differentially expressed after activation of CREB signaling (Pardo et al., 2017), suggesting that CREB signaling may be an important mediator of astrocytic responses to stimuli. To examine this possibility, it is important to identify upstream elements that activate the CREB pathway and downstream pathways that are affected by the CREB pathway. Therefore, this review summarizes astrocytic receptors and transcriptomic and morphological changes related to CREB signaling.

### Neuronal CREB in synaptic communication and plasticity

Early studies of synaptic plasticity have shown CREB to be a crucial mediator of long-term potentiation (LTP). In Aplysia, serotonin (5-HT)-induced long-term facilitation (LTF) of synapses activates the cAMP second messenger pathway and phosphorylates CREB (Kaang et al., 1993; Lee et al., 2009). Subsequently, other studies in Drosophila (Yin et al., 1994) and rodents (Bourtchuladze et al., 1994; Guzowski and McGaugh, 1997) demonstrated that blockade of CREB can impair long-term memory, confirming that CREB is an important mediator of long-term synaptic plasticity and memory. In the 2000s, CREB studies were expanded using transgenic mice expressing constitutively active CREB, called VP16-CREB (Barco et al., 2002) or region-specific modulation using viral expression (Josselyn et al., 2001). VP16-CREB mice show facilitated LTP in the hippocampus (Barco et al., 2002), which is dependent on brain-derived neurotrophic factor (BDNF) expression (Barco et al., 2005). Virus-mediated CREB overexpression in the amygdala can increase long-term memory (Josselyn et al., 2001). Viral modulation of CREB expression has enabled more complex experiments that provide an extensive understanding of CREB. Han et al. (2007) showed that CREB-expressed subset of neurons can act as memory-encoding neurons, which was later confirmed by inhibition of memory by selective ablation (Han et al., 2009). In addition, CREB not only regulates plasticity during synaptic activity but also regulates the excitability of neurons (Zhou et al., 2009).

With increasing evidence of CREB being an important mediator of synaptic communication, the mechanism of CREB activation in neurons has also been investigated. Ser-133 is an important phosphorylation site for CREB activation (Gonzalez and Montminy, 1989). Phosphorylation of CREB at Ser-133 allows binding of CREB-binding protein to the site, inducing a complex formation (Parker et al., 1996). In neurons, various kinases have been shown to be involved in phosphorylation of CREB (for review see Sakamoto et al., 2011). cAMP and PKA are major activators of CREB (Gonzalez and Montminy, 1989) while Akt is required for some CREB activation pathways (Brami-Cherrier et al., 2002), and calcium-dependent pathways involving Ca^2+^/calmodulin (CaM) kinases are also responsible for CREB activation (Deisseroth et al., 1996; Finkbeiner et al., 1997). CREB can be regulated by mechanisms other than phosphorylation, which have been described previously (for review, see Sakamoto et al., 2011).

### G protein-coupled receptors and cyclic adenosine monophosphate/protein kinase A pathways in astrocytic CREB activation

Identifying the signals and receptors that activate CREB would be the first step in understanding the synaptic communication of the tripartite synapse. Unlike neurons, in astrocytes, CREB transcription is not merely induced by Ca^2+^ transients (Murray et al., 2009), thus, emphasizing the need to identify specific receptors that activate distinct pathways for CREB activation. The G protein-coupled receptor (GPCR)-activated cAMP/PKA pathway is the best-known pathway for CREB activation. Therefore, in this section, we explore the current literature on cAMP/PKA/CREB pathways.

#### G protein-coupled receptors that induce response to synaptic signals

G protein-coupled receptors are membrane receptors that bind ligands in the extracellular domain and G proteins in the intracellular domain. Upon activation, GPCRs recruit and activate G proteins, which mediate subsequent activation cascades. GPCRs can be classified according to the type of G proteins they bind to. Canonical cAMP-PKA-CREB signaling can be activated by G_*s*_-GPCRs, where the activated G_*s*_-protein stimulates the production of cAMP, which in turn activates PKA, which can directly phosphorylate CREB. Various types of GPCR that respond to neurotransmitters are present in astrocytes.

One of the known GPCRs that can evoke astrocytic response is the adenosine receptor. All four types of the adenosine receptors, namely A1, A2A, A2B, and A3, are expressed in astrocytes (Dare et al., 2007). A1 and A3 receptors are G_*i*_-coupled, and A2A and A2B receptors are G_*s*_-coupled (Fredholm et al., 2000). The ligand of the receptor adenosine is generally produced through the breakdown of adenosine triphosphate (ATP) by ectoenzymes. The application of ATP (Perea and Araque, 2007; Kawamura and Kawamura, 2011), adenosine (Tanaka et al., 2021), and A2A receptor agonists (Kanno and Nishizaki, 2012) have been reported to evoke astrocytic calcium responses. The calcium response is reduced by A1, A2A, and A2B antagonists (Tanaka et al., 2021). Adenosine has been shown to be involved in controlling neurotransmitter concentrations in astrocytes. Activation of the A2A receptor inhibits glutamate clearance into astrocytes (Nishizaki et al., 2002; Matos et al., 2013) and promotes glutamate release (Nishizaki et al., 2002). In addition, A2A receptor activation boosts γ-aminobutyric acid (GABA) uptake into astrocytes, whereas activation of the A1A receptor depresses GABA uptake by astrocytes (Cristovao-Ferreira et al., 2013). Another G_*s*_-coupled adenosine receptor, the A2B receptor, is involved in the downregulation of mGluR5 receptors and a decrease in excitatory synapses during development (Tanaka et al., 2021). Knockout of the A2A receptor has been shown to enhance memory (Orr et al., 2015). These studies confirm that astrocytes respond to adenosine signaling in a diverse and complex manner, the effects of which are specific to the types of GPCR. Indeed, the behavioral consequences of activating hippocampal astrocytes by G_*q*_- (Adamsky et al., 2018) and G_*i*_- (Kol et al., 2020) coupled designer receptors exclusively activated by designer drugs (DREADDs) are different.

Adrenergic receptors can induce calcium transients in astrocytes (Vardjan and Zorec, 2017; Fischer et al., 2021). All adrenergic receptor (AR) types are expressed in astrocytes (Hertz et al., 2010), and have been studied in various aspects of cognitive function, including consolidation of memory (Gibbs and Bowser, 2010; Gao et al., 2016). α1-ARs are G_*q*_-coupled, α2-ARs are G_*i*_-coupled, and β-ARs are G_*s*_-coupled (Insel, 1993), suggesting that each subtype shows different downstream pathways. Indeed, in olfactory bulbs, only α1-AR and α2-AR are involved in the norepinephrine-induced astrocytic calcium transient, whereas β-AR does not participate in calcium activity (Fischer et al., 2021). Instead, G_*s*_-coupled β1-AR has been shown to induce CREB phosphorylation and cAMP production in neurons (Meitzen et al., 2011). Norepinephrine has also been shown to be involved in astrocytic formation. While activation of β-AR promotes process formation, activation of α2-AR inhibits the β-AR effect (Kitano et al., 2021). In addition, β-AR is involved in glial fibrillary acidic protein (GFAP) plasticity in the soma and processes of astrocytes (Wang et al., 2017). It is unknown whether CREB mediates norepinephrine-regulated astrocytic morphological changes; therefore, future studies are required to evaluate these changes. In addition to morphological control, the activation of ARs can affect glucose uptake in astrocytes (Catus et al., 2011).

#### G protein-coupled receptor-induced canonical cyclic adenosine monophosphate/protein kinase A pathway and astrocytic CREB activation

The CREB pathway has been studied as a downstream pathway of adenosine and adrenergic signaling in astrocytes. Norepinephrine and ATP increase CREB-dependent signaling in astrocytes (Carriba et al., 2012). There is evidence that adrenergic and adenosine receptors activate the cAMP-PKA-CREB pathway in astrocytes; inhibiting PKA blocked adenosine-induced gliotransmitter release in astrocytes (Nishizaki et al., 2002). In addition, PKA blockade inhibits modulation of GABA uptake by the A2A receptor and A1A receptor (Cristovao-Ferreira et al., 2013), suggesting that the astrocytic response to adenosine involves the PKA pathway. Norepinephrine has been shown to induce BDNF in a CREB-dependent manner (Koppel et al., 2018). Transcriptional changes induced by CREB manipulation provide further evidence that the adrenergic receptor pathway is involved in CREB activation. Pardo et al. (2017) expressed constitutively active CREB, VP16-CREB, in astrocytes and compared their transcriptional profiles with norepinephrine- and forskolin-treated astrocytes. Transcription profiles related to amino acid processing, cytoskeleton dynamics, and vesicle dynamics undergo alterations in VP16-CREB cells, similar to the transcriptional profiles induced by norepinephrine and forskolin.

#### G protein-coupled receptor-induced CREB and synaptic plasticity

The cAMP/PKA pathway is a canonical pathway that activates CREB. Zhou et al. (2021) showed that astrocytic cAMP was sufficient to modulate memory and synaptic plasticity. The study used photoactivatable adenylyl cyclase to increase cAMP levels in astrocytes during blue light irradiation, which successfully increased pCREB in target astrocytes. Light stimulation during and immediately after learning significantly increased learning, whereas light stimulation during the retention period impaired memory. These results show that CREB signaling in astrocytes is important for learning, and that its activated time window is important for accurate memory processing. The same study showed that the activation of the cAMP pathway was sufficient to induce *de novo* synaptic plasticity. This is consistent with the results of Adamsky et al. (2018), who showed that G_*q*_-DREADD-mediated astrocytic activation resulted in *de novo* synaptic plasticity. It is possible that the CREB pathway in astrocytes mediates *de novo* synaptic plasticity during learning.

### Non-canonical pathways

Non-canonical CREB activation is known to be mediated by various cascades, such as ERK, MSK, GSK, p90RSK, PI3K/Akt, CaMKII, and CaMKIV (for review, see Sakamoto et al., 2011; Wang et al., 2018). Indeed, one study reported that ATP- and norepinephrine-induced CREB activation in astrocytes is not mediated by the canonical cAMP pathway, but by protein kinase C (PKC) in culture conditions (Carriba et al., 2012). In addition, various receptors other than GPCR may directly activate CREB or may affect CREB *via* crosstalk between signaling pathways in astrocytes. This section discusses the receptor tyrosine kinase, Notch signaling, and PI3K/Akt pathways.

#### Receptor tyrosine kinases (RTKs)

Receptor tyrosine kinases are membrane proteins with an intracellular tyrosine kinase domain, which activates various downstream molecules that can regulate the CREB pathway. Various receptor tyrosine kinases that mediate synaptic communication are expressed in astrocytes, such as the IGF1R, which has been shown to induce calcium responses, ATP release, and affect synaptic plasticity (Noriega-Prieto et al., 2021). Astrocytic IGF1R modulates PTEN, which is involved in the PI3K/Akt pathway (Fernandez et al., 2008). Another type of receptor tyrosine kinase expressed in astrocytes, the ephrin receptors (Zhuang et al., 2010), have been reported to regulate the astrocytic cytoskeleton (Puschmann and Turnley, 2010) and is involved in gliotransmitter release (Zhuang et al., 2010). Ephrin signaling is known to induce CREB activation in neurons (Alapin et al., 2018; Yuan et al., 2021); however, there is a lack of direct evidence of its involvement in astrocytic ephrin receptors and CREB. Instead, the astrocytic ephrin receptor has been reported to modulate PKC (Zhuang et al., 2010), which may be related to CREB-binding protein (CBP) interaction (Johnson et al., 2007). RTK may transactivate other types of receptors that activate CREB pathway. In PC12 cells, TrkA receptor transactivates sustained CREB activation by α2-AR in GPCR kinase 2 (GRK2)-dependent fashion (Karkoulias et al., 2020). GRK2 is known to mediate crosstalk between RTKs and GPCRs (Fu et al., 2017). GRK2 is expressed in astrocyte, known to regulate glutamate transport (Nijboer et al., 2013).

#### Notch signaling

Notch signaling interacts with and represses the CREB pathway (Hallaq et al., 2015). It is a juxtracrine signaling system, mostly between membrane-bound ligands and receptors of adjacent cells. It can be mediated by the cleavage of the intracellular domain, and has been shown to induce CREB activation in the brain and is involved in long-term memory (Zhang et al., 2013). Heterogeneous Notch signaling molecules, including Notch1, Bmp4, Hes1, and Nrarp, are expressed in astrocytes (Hu et al., 2019). Expression of NICD were shown to be involved in CREB activation *via* MAPK in astroglia culture (Lim et al., 2019). While neuronal Notch signaling is known to be activity-responsive and necessary for learning (Costa et al., 2003; Alberi et al., 2011), little is known about the astrocyte-neuron communication. Astrocytic Notch signaling has mainly been studied for its role in development and differentiation. Considering that astrocytes are in close proximity to neurons, Notch signaling could also be a potential mediator of contact-dependent cell-to-cell communication in tripartite synapses. An *in vitro* study showed that an astrocyte-neuron co-culture differentially expressed Notch signaling pathway-related genes, which were blocked by a Notch pathway inhibitor (Hasel et al., 2017). In addition, *ex vivo* hippocampal astrocytes showed differential expression of Nrarp after late LTP (Chen et al., 2017), suggesting the effect of neuronal activity and synaptic communication on astrocytic Notch signaling. Many studies have reported the pathological involvement of astrocytic Notch signaling, which is activated in reactive astrocytes (Ribeiro et al., 2021), and the Notch ligand of reactive astrocytes has been shown to mediate symptoms in a neurodegenerative model (Nonneman et al., 2018). The NFIA transcription factor, which stimulates basal transcription, is known to be activated downstream of Notch signaling in astrocytes during development (Namihira et al., 2009); however, it is not known if a similar downstream system is present in the adult brain. NFIA can interact with CREB indirectly *via* CBP (Leahy et al., 1999), which may be the mechanism of CREB repression by Notch signaling. In addition, PKA signaling is known to regulate expression of Notch (Angulo-Rojo et al., 2013), suggesting bidirectional involvement between pathways.

#### Phosphatidylinositol 3-kinase pathway

The PI3K/Akt pathway is involved in cellular proliferation and survival. The activation of PI3K-Akt signaling is known to activate CREB downstream and is involved in brain function. In the PI3K pathway, activated receptors stimulate PI3Ks, which in turn mediate the conversion of 4,5-bisphosphate (PIP_2_) to phosphatidylinositol 3,4,5-trisphosphate (PIP_3_), leading to Akt activation (Hemmings and Restuccia, 2012). Akt is involved in multiple signaling pathways, including CREB, where it mediates fibroblast growth factor (FGF)-2-induced activation (Peltier et al., 2007). In addition, alcohol withdrawal activates the PI3K-Akt-CREB pathway (Qiao et al., 2018). Receptors that activate the PI3K/Akt pathway include receptor tyrosine kinase and GPCR, depending on the PI3K isoforms. Notch signaling is known to affect PTEN (Palomero et al., 2007; Serra et al., 2015), which inhibits the PI3K/Akt pathway. In astrocytes, stimulation of the melatonin receptor, a type of GPCR, activates CREB *via* the Akt pathway (Kong et al., 2008).

#### Ion channels

Intracellular calcium signaling is involved in CREB activation in neurons (Chawla et al., 1998; Hardingham et al., 2001), and calcium-induced activation in turn is mediated by CaM kinases (Deisseroth et al., 1996; Finkbeiner et al., 1997). However, in astrocytes, increasing calcium itself does not induce CREB activation (Murray et al., 2009).

### Transcriptional effect of synaptic communication and CREB activation in astrocyte

Molecular pathways that mediate astrocyte-neuron communication may be activated by neuron-released or contact-dependent factors, induce calcium signals, to regulate transcription. A few studies have analyzed activity-induced differential gene expression in astrocytes. Neuronal activity can induce Nuclear factor erythroid 2-related factor 2 (Nfr2) pathway in astrocytes in a calcium-dependent manner (Habas et al., 2013), which has two CBP binding sites that are crucial for its activity (Katoh et al., 2001). Nrf2 is involved in the upregulation of glutathione metabolism gene expression (McGann and Mandel, 2018), which is known to be involved in protection against oxidative stress, and CREB-dependent transcription is observed in astrocytes after neuronal activation, upregulating glucose and lactate metabolism, and lactate shuttle-related genes (Hasel et al., 2017). The neuron-astrocyte lactate shuttle is required for learning (Suzuki et al., 2011), and its inhibition blocks learning-induced mRNA translation in neurons (Descalzi et al., 2019). In addition, transcriptional changes in astrocytes after learning, which are natural conditions that elicit neuronal activity, have been reported. After inhibitory avoidance training, synaptic function-related genes are differentially expressed in astrocytes, including NadK2 upregulation (Katzman et al., 2021).

Gene expression by direct manipulation of the CREB transcription factor will further explain the importance of astrocytic participation in synaptic activity and cognitive function. Various transcriptional changes were induced by CREB activation in astrocytes (Pardo et al., 2017). BDNF is one of the proteins that are upregulated by CREB-dependent pathway in astrocytes (Koppel et al., 2018). Astrocytes express both BDNF (Saha et al., 2006; Koppel et al., 2018) and TrkB (Holt et al., 2019), which have been widely studied for their neuroprotective properties in epilepsy (Fernandez-Garcia et al., 2020), Alzheimer’s disease (de Pins et al., 2019), and Huntington’s disease (Hong et al., 2016).

Although CREB may or may not be activated by calcium signaling itself, CREB activity may modulate astrocytic calcium signaling. With regard to astrocytic calcium signaling, a study by Eraso-Pichot et al. (2017) showed that activating CREB signaling by transmitters and VP16-CREB reduced cytosolic astrocytic calcium by upregulating the sigma-1 receptor that participates in endoplasmic reticulum calcium transfer.

### CREB in reactive astrocyte and pathology

The CREB transcription factor is involved in various neurodegenerative disorders, including Alzheimer’s disease. Reactive astrocytes have recently gained attention as a cause of neurodegenerative diseases, and CREB expression in reactive astrocytes has been reported to be neuroprotective (Pardo et al., 2016). High GFAP expression, a reporter of astrocytic reactivity, is inversely associated with CREB content, as shown in a neurodegenerative disease model (Pugazhenthi et al., 2011). MAO-B is another enzyme involved in reactive astrogliosis (Chun et al., 2022) and neurodegeneration (Mallajosyula et al., 2008), and MAO-B promoter is known to be regulated by the CREB transcription factor (Arige et al., 2019). Astrocytic CREB is activated during melatonin-induced neuroprotection (Kong et al., 2008), and is also involved in inflammatory conditions. Astrocytes produce chemokines in response to CCL5, which is mediated by the CREB activity (Zhang et al., 2002).

## Conclusion

We summarize various types of receptors and pathways involved in synaptic communication that may activate CREB in astrocytes and briefly discusses the consequences of CREB activation. Adenosine and adrenergic receptors are GPCRs mainly studied for neuron-astrocyte communication and subsequent CREB activation, which is mediated by the cAMP-PKA pathway. Although other types of receptors, such as RTKs and Notch receptors, have been documented, these studies have scarcely focused on the CREB pathway. Different receptors are involved in separate downstream pathways; however, it is yet to be determined whether they participate in CREB activation. Finally, this review summarizes CREB-mediated gene expression in astrocytes. It is to be noted that only the genes that are known to be important for astrocytic and synaptic functions were selected for discussion, and there are many more genes regulated by CREB activation involving complex mechanisms, which should be examined thoroughly. In summary, the evidence presented in this mini-review suggests the importance of CREB activation in astrocytic communication with neuronal synapses and the possible pathways that mediate such effects.

## Author contributions

JK wrote the initial draft in consultation with B-KK. JK and B-KK reviewed and edited the manuscript. Both authors contributed to the writing of this review manuscript.

## References

[B1] Adamsky A. Kol A. Kreisel T. Doron A. Ozeri-Engelhard N. Melcer T. (2018). Astrocytic activation generates de novo neuronal potentiation and memory enhancement. *Cell* 174 59–71e14. 10.1016/j.cell.2018.05.002 29804835

[B2] Alapin J. M. Dines M. Vassiliev M. Tamir T. Ram A. Locke C. (2018). Activation of EphB2 forward signaling enhances memory consolidation. *Cell Rep.* 23 2014–2025. 10.1016/j.celrep.2018.04.042 29768201 PMC6314675

[B3] Alberi L. Liu S. Wang Y. Badie R. Smith-Hicks C. Wu J. (2011). Activity-induced Notch signaling in neurons requires Arc/Arg3.1 and is essential for synaptic plasticity in hippocampal networks. *Neuron* 69 437–444. 10.1016/j.neuron.2011.01.004 21315255 PMC3056341

[B4] Angulo-Rojo C. Manning-Cela R. Aguirre A. Ortega A. Lopez-Bayghen E. (2013). Involvement of the Notch pathway in terminal astrocytic differentiation: Role of PKA. *ASN Neuro* 5:e00130. 10.1042/AN20130023 24286475 PMC3891361

[B5] Arige V. Agarwal A. Khan A. A. Kalyani A. Natarajan B. Gupta V. (2019). Regulation of monoamine oxidase b gene expression: Key roles for transcription factors Sp1, Egr1 and CREB, and microRNAs miR-300 and miR-1224. *J. Mol. Biol.* 431 1127–1147. 10.1016/j.jmb.2019.01.042 30738894

[B6] Barco A. Alarcon J. M. Kandel E. R. (2002). Expression of constitutively active CREB protein facilitates the late phase of long-term potentiation by enhancing synaptic capture. *Cell* 108 689–703. 10.1016/s0092-8674(02)00657-8 11893339

[B7] Barco A. Patterson S. L. Alarcon J. M. Gromova P. Mata-Roig M. Morozov A. (2005). Gene expression profiling of facilitated L-LTP in VP16-CREB mice reveals that BDNF is critical for the maintenance of LTP and its synaptic capture. *Neuron* 48 123–137. 10.1016/j.neuron.2005.09.005 16202713

[B8] Bourtchuladze R. Frenguelli B. Blendy J. Cioffi D. Schutz G. Silva A. J. (1994). Deficient long-term memory in mice with a targeted mutation of the cAMP-responsive element-binding protein. *Cell* 79 59–68. 10.1016/0092-8674(94)90400-6 7923378

[B9] Brami-Cherrier K. Valjent E. Garcia M. Pages C. Hipskind R. A. Caboche J. (2002). Dopamine induces a PI3-kinase-independent activation of Akt in striatal neurons: A new route to cAMP response element-binding protein phosphorylation. *J. Neurosci.* 22 8911–8921. 10.1523/JNEUROSCI.22-20-08911.2002 12388598 PMC6757682

[B10] Bushong E. A. Martone M. E. Jones Y. Z. Ellisman M. H. (2002). Protoplasmic astrocytes in CA1 stratum radiatum occupy separate anatomical domains. *J. Neurosci.* 22 183–192. 10.1523/JNEUROSCI.22-01-00183.2002 11756501 PMC6757596

[B11] Carriba P. Pardo L. Parra-Damas A. Lichtenstein M. P. Saura C. A. Pujol A. (2012). ATP and noradrenaline activate CREB in astrocytes via noncanonical Ca(2+) and cyclic AMP independent pathways. *Glia* 60 1330–1344. 10.1002/glia.22352 22593004

[B12] Catus S. L. Gibbs M. E. Sato M. Summers R. J. Hutchinson D. S. (2011). Role of beta-adrenoceptors in glucose uptake in astrocytes using beta-adrenoceptor knockout mice. *Br. J. Pharmacol.* 162 1700–1715. 10.1111/j.1476-5381.2010.01153.x 21138422 PMC3081115

[B13] Chawla S. Hardingham G. E. Quinn D. R. Bading H. (1998). CBP: A signal-regulated transcriptional coactivator controlled by nuclear calcium and CaM kinase IV. *Science* 281 1505–1509. 10.1126/science.281.5382.1505 9727976

[B14] Chen P. B. Kawaguchi R. Blum C. Achiro J. M. Coppola G. O’Dell T. J. (2017). Mapping gene expression in excitatory neurons during hippocampal late-phase long-term potentiation. *Front. Mol. Neurosci.* 10:39. 10.3389/fnmol.2017.00039 28275336 PMC5319997

[B15] Chun H. Lim J. Park K. D. Lee C. J. (2022). Inhibition of monoamine oxidase B prevents reactive astrogliosis and scar formation in stab wound injury model. *Glia* 70 354–367. 10.1002/glia.24110 34713936

[B16] Costa R. M. Honjo T. Silva A. J. (2003). Learning and memory deficits in notch mutant mice. *Curr. Biol.* 13 1348–1354. 10.1016/s0960-9822(03)00492-5 12906797

[B17] Cristovao-Ferreira S. Navarro G. Brugarolas M. Perez-Capote K. Vaz S. H. Fattorini G. (2013). A1R-A2AR heteromers coupled to Gs and G i/0 proteins modulate GABA transport into astrocytes. *Purinergic Signal.* 9 433–449. 10.1007/s11302-013-9364-5 23657626 PMC3757138

[B18] Dare E. Schulte G. Karovic O. Hammarberg C. Fredholm B. B. (2007). Modulation of glial cell functions by adenosine receptors. *Physiol. Behav.* 92 15–20. 10.1016/j.physbeh.2007.05.031 17574632

[B19] de Pins B. Cifuentes-Diaz C. Farah A. T. Lopez-Molina L. Montalban E. Sancho-Balsells A. (2019). Conditional BDNF delivery from astrocytes rescues memory deficits, spine density, and synaptic properties in the 5xFAD mouse model of Alzheimer disease. *J. Neurosci.* 39 2441–2458. 10.1523/JNEUROSCI.2121-18.2019 30700530 PMC6435824

[B20] Deisseroth K. Bito H. Tsien R. W. (1996). Signaling from synapse to nucleus: Postsynaptic CREB phosphorylation during multiple forms of hippocampal synaptic plasticity. *Neuron* 16 89–101. 10.1016/s0896-6273(00)80026-4 8562094

[B21] Descalzi G. Gao V. Steinman M. Q. Suzuki A. Alberini C. M. (2019). Lactate from astrocytes fuels learning-induced mRNA translation in excitatory and inhibitory neurons. *Commun. Biol.* 2:247. 10.1038/s42003-019-0495-2 31286064 PMC6606643

[B22] Eraso-Pichot A. Larramona-Arcas R. Vicario-Orri E. Villalonga R. Pardo L. Galea E. (2017). CREB decreases astrocytic excitability by modifying subcellular calcium fluxes via the sigma-1 receptor. *Cell Mol. Life Sci.* 74 937–950. 10.1007/s00018-016-2397-5 27761593 PMC11107612

[B23] Fernandez S. Garcia-Garcia M. Torres-Aleman I. (2008). Modulation by insulin-like growth factor I of the phosphatase PTEN in astrocytes. *Biochim. Biophys. Acta* 1783 803–812. 10.1016/j.bbamcr.2007.10.020 18062928

[B24] Fernandez-Garcia S. Sancho-Balsells A. Longueville S. Herve D. Gruart A. Delgado-Garcia J. M. (2020). Astrocytic BDNF and TrkB regulate severity and neuronal activity in mouse models of temporal lobe epilepsy. *Cell Death Dis.* 11:411. 10.1038/s41419-020-2615-9 32483154 PMC7264221

[B25] Finkbeiner S. Tavazoie S. F. Maloratsky A. Jacobs K. M. Harris K. M. Greenberg M. E. (1997). CREB: A major mediator of neuronal neurotrophin responses. *Neuron* 19 1031–1047. 10.1016/s0896-6273(00)80395-5 9390517

[B26] Fischer T. Prey J. Eschholz L. Rotermund N. Lohr C. (2021). Norepinephrine-induced calcium signaling and store-operated calcium entry in olfactory bulb astrocytes. *Front. Cell Neurosci.* 15:639754. 10.3389/fncel.2021.639754 33833669 PMC8021869

[B27] Fredholm B. B. Arslan G. Halldner L. Kull B. Schulte G. Wasserman W. (2000). Structure and function of adenosine receptors and their genes. *Naunyn Schmiedebergs Arch Pharmacol.* 362 364–374. 10.1007/s002100000313 11111830

[B28] Fu Q. Shi Q. West T. M. Xiang Y. K. (2017). Cross-talk between insulin signaling and G protein-coupled receptors. *J. Cardiovasc. Pharmacol.* 70 74–86. 10.1097/FJC.0000000000000481 28328746 PMC5734060

[B29] Gao V. Suzuki A. Magistretti P. J. Lengacher S. Pollonini G. Steinman M. Q. (2016). Astrocytic beta2-adrenergic receptors mediate hippocampal long-term memory consolidation. *Proc. Natl. Acad. Sci. U.S.A.* 113 8526–8531. 10.1073/pnas.1605063113 27402767 PMC4968707

[B30] Gibbs M. E. Bowser D. N. (2010). Astrocytic adrenoceptors and learning: Alpha1-adrenoceptors. *Neurochem. Int.* 57 404–410. 10.1016/j.neuint.2010.03.020 20380858

[B31] Gonzalez G. A. Montminy M. R. (1989). Cyclic AMP stimulates somatostatin gene transcription by phosphorylation of CREB at serine 133. *Cell* 59 675–680. 10.1016/0092-8674(89)90013-5 2573431

[B32] Guzowski J. F. McGaugh J. L. (1997). Antisense oligodeoxynucleotide-mediated disruption of hippocampal cAMP response element binding protein levels impairs consolidation of memory for water maze training. *Proc. Natl. Acad. Sci. U.S.A.* 94 2693–2698. 10.1073/pnas.94.6.2693 9122258 PMC20151

[B33] Habas A. Hahn J. Wang X. Margeta M. (2013). Neuronal activity regulates astrocytic Nrf2 signaling. *Proc. Natl. Acad. Sci. U.S.A.* 110 18291–18296. 10.1073/pnas.1208764110 24145448 PMC3831500

[B34] Hallaq R. Volpicelli F. Cuchillo-Ibanez I. Hooper C. Mizuno K. Uwanogho D. (2015). The Notch intracellular domain represses CRE-dependent transcription. *Cell Signal.* 27 621–629. 10.1016/j.cellsig.2014.11.034 25479589

[B35] Han J. H. Kushner S. A. Yiu A. P. Cole C. J. Matynia A. Brown R. A. (2007). Neuronal competition and selection during memory formation. *Science* 316 457–460. 10.1126/science.1139438 17446403

[B36] Han J. H. Kushner S. A. Yiu A. P. Hsiang H. L. Buch T. Waisman A. (2009). Selective erasure of a fear memory. *Science* 323 1492–1496. 10.1126/science.1164139 19286560

[B37] Hardingham G. E. Arnold F. J. Bading H. (2001). Nuclear calcium signaling controls CREB-mediated gene expression triggered by synaptic activity. *Nat. Neurosci.* 4 261–267. 10.1038/85109 11224542

[B38] Hasel P. Dando O. Jiwaji Z. Baxter P. Todd A. C. Heron S. (2017). Neurons and neuronal activity control gene expression in astrocytes to regulate their development and metabolism. *Nat. Commun.* 8:15132. 10.1038/ncomms15132 28462931 PMC5418577

[B40] Hemmings B. A. Restuccia D. F. (2012). PI3K-PKB/Akt pathway. *Cold Spring Harb Perspect. Biol.* 4:a011189. 10.1101/cshperspect.a011189 22952397 PMC3428770

[B39] Hertz L. Lovatt D. Goldman S. A. Nedergaard M. (2010). Adrenoceptors in brain: Cellular gene expression and effects on astrocytic metabolism and [Ca(2+)]i. *Neurochem. Int.* 57 411–420. 10.1016/j.neuint.2010.03.019 20380860 PMC2934885

[B41] Holt L. M. Hernandez R. D. Pacheco N. L. Torres Ceja B. Hossain M. Olsen M. L. (2019). Astrocyte morphogenesis is dependent on BDNF signaling via astrocytic TrkB.T1. *Elife* 8:e44667. 10.7554/eLife.44667 31433295 PMC6726422

[B42] Hong Y. Zhao T. Li X. J. Li S. (2016). Mutant huntingtin impairs BDNF release from astrocytes by disrupting conversion of Rab3a-GTP into Rab3a-GDP. *J. Neurosci.* 36 8790–8801. 10.1523/JNEUROSCI.0168-16.2016 27559163 PMC4995297

[B43] Hu X. Qin S. Huang X. Yuan Y. Tan Z. Gu Y. (2019). Region-restrict astrocytes exhibit heterogeneous susceptibility to neuronal reprogramming. *Stem. Cell Rep.* 12 290–304. 10.1016/j.stemcr.2018.12.017 30713039 PMC6373495

[B44] Insel P. A. (1993). Adrenergic receptors, G proteins, and cell regulation: Implications for aging research. *Exp. Gerontol.* 28 341–348. 10.1016/0531-5565(93)90061-h 8224033

[B45] Johnson J. Albarani V. Nguyen M. Goldman M. Willems F. Aksoy E. (2007). Protein kinase Calpha is involved in interferon regulatory factor 3 activation and type I interferon-beta synthesis. *J. Biol. Chem.* 282 15022–15032. 10.1074/jbc.M700421200 17296604

[B46] Josselyn S. A. Shi C. Carlezon W. A. Jr. Neve R. L. Nestler E. J. Davis M. (2001). Long-term memory is facilitated by cAMP response element-binding protein overexpression in the amygdala. *J. Neurosci.* 21 2404–2412. 10.1523/JNEUROSCI.21-07-02404.2001 11264314 PMC6762400

[B47] Kaang B. K. Kandel E. R. Grant S. G. (1993). Activation of cAMP-responsive genes by stimuli that produce long-term facilitation in Aplysia sensory neurons. *Neuron* 10 427–435. 10.1016/0896-6273(93)90331-k 8384857

[B48] Kanno T. Nishizaki T. (2012). A(2a) adenosine receptor mediates PKA-dependent glutamate release from synaptic-like vesicles and Ca(2+) efflux from an IP(3)- and ryanodine-insensitive intracellular calcium store in astrocytes. *Cell Physiol. Biochem.* 30 1398–1412. 10.1159/000343328 23154210

[B49] Karkoulias G. McCrink K. A. Maning J. Pollard C. M. Desimine V. L. Patsouras N. (2020). Sustained GRK2-dependent CREB activation is essential for alpha2-adrenergic receptor-induced PC12 neuronal differentiation. *Cell Signal.* 66:109446. 10.1016/j.cellsig.2019.109446 31678682

[B50] Katoh Y. Itoh K. Yoshida E. Miyagishi M. Fukamizu A. Yamamoto M. (2001). Two domains of Nrf2 cooperatively bind CBP, a CREB binding protein, and synergistically activate transcription. *Genes Cells* 6 857–868. 10.1046/j.1365-2443.2001.00469.x 11683914

[B51] Katzman A. Khodadadi-Jamayran A. Kapeller-Libermann D. Ye X. Tsirigos A. Heguy A. (2021). Distinct transcriptomic profiles in the dorsal hippocampus and prelimbic cortex are transiently regulated following episodic learning. *J. Neurosci.* 41 2601–2614. 10.1523/JNEUROSCI.1557-20.2021 33536202 PMC8018728

[B52] Kawamura M. Jr. Kawamura M. (2011). Long-term facilitation of spontaneous calcium oscillations in astrocytes with endogenous adenosine in hippocampal slice cultures. *Cell Calcium* 49 249–258. 10.1016/j.ceca.2011.02.009 21402407

[B53] Kitano T. Eguchi R. Okamatsu-Ogura Y. Yamaguchi S. Otsuguro K. I. (2021). Opposing functions of alpha- and beta-adrenoceptors in the formation of processes by cultured astrocytes. *J. Pharmacol. Sci.* 145 228–240. 10.1016/j.jphs.2020.12.005 33602503

[B54] Kol A. Adamsky A. Groysman M. Kreisel T. London M. Goshen I. (2020). Astrocytes contribute to remote memory formation by modulating hippocampal-cortical communication during learning. *Nat. Neurosci.* 23 1229–1239. 10.1038/s41593-020-0679-6 32747787 PMC7611962

[B55] Kong P. J. Byun J. S. Lim S. Y. Lee J. J. Hong S. J. Kwon K. J. (2008). Melatonin Induces akt phosphorylation through melatonin receptor- and PI3K-dependent pathways in primary astrocytes. *Korean J. Physiol. Pharmacol.* 12 37–41. 10.4196/kjpp.2008.12.2.37 20157392 PMC2817532

[B56] Koppel I. Jaanson K. Klasche A. Tuvikene J. Tiirik T. Parn A. (2018). Dopamine cross-reacts with adrenoreceptors in cortical astrocytes to induce BDNF expression, CREB signaling and morphological transformation. *Glia* 66 206–216. 10.1002/glia.23238 28983964

[B57] Leahy P. Crawford D. R. Grossman G. Gronostajski R. M. Hanson R. W. (1999). CREB binding protein coordinates the function of multiple transcription factors including nuclear factor I to regulate phosphoenolpyruvate carboxykinase (GTP) gene transcription. *J. Biol. Chem.* 274 8813–8822. 10.1074/jbc.274.13.8813 10085123

[B58] Lee Y. S. Choi S. L. Lee S. H. Kim H. Park H. Lee N. (2009). Identification of a serotonin receptor coupled to adenylyl cyclase involved in learning-related heterosynaptic facilitation in Aplysia. *Proc. Natl. Acad. Sci. U.S.A.* 106 14634–14639. 10.1073/pnas.0907502106 19706550 PMC2732834

[B59] Lezmy J. Arancibia-Carcamo I. L. Quintela-Lopez T. Sherman D. L. Brophy P. J. Attwell D. (2021). Astrocyte Ca(2+)-evoked ATP release regulates myelinated axon excitability and conduction speed. *Science* 374:eabh2858. 10.1126/science.abh2858 34648330 PMC7611967

[B60] Lim H. J. Park J. H. Jo C. Yoon K. Koh Y. H. (2019). Cigarette smoke extracts and cadmium induce COX-2 expression through gamma-secretase-mediated p38 MAPK activation in C6 astroglia cells. *PLoS One* 14:e0212749. 10.1371/journal.pone.0212749 30794693 PMC6386363

[B61] Mallajosyula J. K. Kaur D. Chinta S. J. Rajagopalan S. Rane A. Nicholls D. G. (2008). MAO-B elevation in mouse brain astrocytes results in Parkinson’s pathology. *PLoS One* 3:e1616. 10.1371/journal.pone.0001616 18286173 PMC2229649

[B62] Martin R. Bajo-Graneras R. Moratalla R. Perea G. Araque A. (2015). Circuit-specific signaling in astrocyte-neuron networks in basal ganglia pathways. *Science* 349 730–734. 10.1126/science.aaa7945 26273054

[B63] Martin-Fernandez M. Jamison S. Robin L. M. Zhao Z. Martin E. D. Aguilar J. (2017). Synapse-specific astrocyte gating of amygdala-related behavior. *Nat. Neurosci.* 20 1540–1548. 10.1038/nn.4649 28945222 PMC5903286

[B64] Matos M. Augusto E. Agostinho P. Cunha R. A. Chen J. F. (2013). Antagonistic interaction between adenosine A2A receptors and Na+/K+-ATPase-alpha2 controlling glutamate uptake in astrocytes. *J. Neurosci.* 33 18492–18502. 10.1523/JNEUROSCI.1828-13.2013 24259572 PMC3834055

[B65] McGann J. C. Mandel G. (2018). Neuronal activity induces glutathione metabolism gene expression in astrocytes. *Glia* 66 2024–2039. 10.1002/glia.23455 30043519 PMC6185788

[B66] Meitzen J. Luoma J. I. Stern C. M. Mermelstein P. G. (2011). beta1-Adrenergic receptors activate two distinct signaling pathways in striatal neurons. *J. Neurochem.* 116 984–995. 10.1111/j.1471-4159.2010.07137.x 21143600 PMC3078043

[B67] Murray P. D. Kingsbury T. J. Krueger B. K. (2009). Failure of Ca2+-activated, CREB-dependent transcription in astrocytes. *Glia* 57 828–834. 10.1002/glia.20809 19031446 PMC2669848

[B68] Namihira M. Kohyama J. Semi K. Sanosaka T. Deneen B. Taga T. (2009). Committed neuronal precursors confer astrocytic potential on residual neural precursor cells. *Dev. Cell* 16 245–255. 10.1016/j.devcel.2008.12.014 19217426

[B69] Nijboer C. H. Heijnen C. J. Degos V. Willemen H. L. Gressens P. Kavelaars A. (2013). Astrocyte GRK2 as a novel regulator of glutamate transport and brain damage. *Neurobiol. Dis.* 54 206–215. 10.1016/j.nbd.2012.12.013 23313319 PMC3628971

[B70] Nishizaki T. Nagai K. Nomura T. Tada H. Kanno T. Tozaki H. (2002). A new neuromodulatory pathway with a glial contribution mediated via A(2a) adenosine receptors. *Glia* 39 133–147. 10.1002/glia.10100 12112365

[B71] Nonneman A. Criem N. Lewandowski S. A. Nuyts R. Thal D. R. Pfrieger F. W. (2018). Astrocyte-derived Jagged-1 mitigates deleterious Notch signaling in amyotrophic lateral sclerosis. *Neurobiol. Dis.* 119 26–40. 10.1016/j.nbd.2018.07.012 30010003

[B72] Noriega-Prieto J. A. Maglio L. E. Zegarra-Valdivia J. A. Pignatelli J. Fernandez A. M. Martinez-Rachadell L. (2021). Astrocytic IGF-IRs induce adenosine-mediated inhibitory downregulation and improve sensory discrimination. *J. Neurosci.* 41 4768–4781. 10.1523/JNEUROSCI.0005-21.2021 33911021 PMC8260162

[B73] Orr A. G. Hsiao E. C. Wang M. M. Ho K. Kim D. H. Wang X. (2015). Astrocytic adenosine receptor A2A and Gs-coupled signaling regulate memory. *Nat. Neurosci.* 18 423–434. 10.1038/nn.3930 25622143 PMC4340760

[B74] Palomero T. Sulis M. L. Cortina M. Real P. J. Barnes K. Ciofani M. (2007). Mutational loss of PTEN induces resistance to NOTCH1 inhibition in T-cell leukemia. *Nat. Med.* 13 1203–1210. 10.1038/nm1636 17873882 PMC2600418

[B75] Pardo L. Schluter A. Valor L. M. Barco A. Giralt M. Golbano A. (2016). Targeted activation of CREB in reactive astrocytes is neuroprotective in focal acute cortical injury. *Glia* 64 853–874. 10.1002/glia.22969 26880229

[B76] Pardo L. Valor L. M. Eraso-Pichot A. Barco A. Golbano A. Hardingham G. E. (2017). CREB regulates distinct adaptive transcriptional programs in astrocytes and neurons. *Sci. Rep.* 7:6390. 10.1038/s41598-017-06231-x 28743894 PMC5526874

[B77] Parker D. Ferreri K. Nakajima T. LaMorte V. J. Evans R. Koerber S. C. (1996). Phosphorylation of CREB at Ser-133 induces complex formation with CREB-binding protein via a direct mechanism. *Mol. Cell Biol.* 16 694–703. 10.1128/MCB.16.2.694 8552098 PMC231049

[B78] Peltier J. O’Neill A. Schaffer D. V. (2007). PI3K/Akt and CREB regulate adult neural hippocampal progenitor proliferation and differentiation. *Dev. Neurobiol.* 67 1348–1361. 10.1002/dneu.20506 17638387

[B79] Perea G. Araque A. (2007). Astrocytes potentiate transmitter release at single hippocampal synapses. *Science* 317 1083–1086. 10.1126/science.1144640 17717185

[B80] Pugazhenthi S. Wang M. Pham S. Sze C. I. Eckman C. B. (2011). Downregulation of CREB expression in Alzheimer’s brain and in Abeta-treated rat hippocampal neurons. *Mol. Neurodegener.* 6:60. 10.1186/1750-1326-6-60 21854604 PMC3174124

[B81] Puschmann T. B. Turnley A. M. (2010). Eph receptor tyrosine kinases regulate astrocyte cytoskeletal rearrangement and focal adhesion formation. *J. Neurochem.* 113 881–894. 10.1111/j.1471-4159.2010.06655.x 20202079

[B82] Qiao X. Gai H. Su R. Deji C. Cui J. Lai J. (2018). PI3K-AKT-GSK3beta-CREB signaling pathway regulates anxiety-like behavior in rats following alcohol withdrawal. *J. Affect. Disord.* 235 96–104. 10.1016/j.jad.2018.04.039 29655081

[B83] Ribeiro T. N. Delgado-Garcia L. M. Porcionatto M. A. (2021). Notch1 and Galectin-3 modulate cortical reactive astrocyte response after brain injury. *Front. Cell Dev. Biol.* 9:649854. 10.3389/fcell.2021.649854 34222228 PMC8244823

[B84] Saha R. N. Liu X. Pahan K. (2006). Up-regulation of BDNF in astrocytes by TNF-alpha: A case for the neuroprotective role of cytokine. *J. Neuroimmune Pharmacol.* 1 212–222. 10.1007/s11481-006-9020-8 18040799 PMC2131740

[B85] Sakamoto K. Karelina K. Obrietan K. (2011). CREB: A multifaceted regulator of neuronal plasticity and protection. *J. Neurochem.* 116 1–9. 10.1111/j.1471-4159.2010.07080.x 21044077 PMC3575743

[B86] Serra H. Chivite I. Angulo-Urarte A. Soler A. Sutherland J. D. Arruabarrena-Aristorena A. (2015). PTEN mediates Notch-dependent stalk cell arrest in angiogenesis. *Nat. Commun.* 6:7935. 10.1038/ncomms8935 26228240 PMC5426521

[B87] Suzuki A. Stern S. A. Bozdagi O. Huntley G. W. Walker R. H. Magistretti P. J. (2011). Astrocyte-neuron lactate transport is required for long-term memory formation. *Cell* 144 810–823. 10.1016/j.cell.2011.02.018 21376239 PMC3073831

[B88] Tanaka M. Shigetomi E. Parajuli B. Nagatomo H. Shinozaki Y. Hirayama Y. (2021). Adenosine A2B receptor down-regulates metabotropic glutamate receptor 5 in astrocytes during postnatal development. *Glia* 69 2546–2558. 10.1002/glia.24006 34339538

[B89] Vardjan N. Zorec R. (2017). *Noradrenergic signaling and astroglia.* San Diego, CA: Elsevier/AP.

[B90] Wang H. Xu J. Lazarovici P. Quirion R. Zheng W. (2018). cAMP response element-binding protein (CREB): A possible signaling molecule link in the pathophysiology of schizophrenia. *Front. Mol. Neurosci.* 11:255. 10.3389/fnmol.2018.00255 30214393 PMC6125665

[B91] Wang P. Qin D. Wang Y. F. (2017). Oxytocin rapidly changes astrocytic GFAP plasticity by differentially modulating the expressions of pERK 1/2 and protein kinase A. *Front. Mol. Neurosci.* 10:262. 10.3389/fnmol.2017.00262 28860967 PMC5559427

[B92] Yin J. C. Wallach J. S. Del Vecchio M. Wilder E. L. Zhou H. Quinn W. G. (1994). Induction of a dominant negative CREB transgene specifically blocks long-term memory in Drosophila. *Cell* 79 49–58. 10.1016/0092-8674(94)90399-9 7923376

[B93] Yuan L. Yi W. Sun C. Ma S. Wang J. Liu S. (2021). EphB2 activates CREB-dependent expression of Annexin A1 to regulate dendritic spine morphogenesis. *Biochem. Biophys. Res. Commun.* 584 107–115. 10.1016/j.bbrc.2021.11.011 34781202

[B94] Zhang J. Little C. J. Tremmel D. M. Yin J. C. Wesley C. S. (2013). Notch-inducible hyperphosphorylated CREB and its ultradian oscillation in long-term memory formation. *J. Neurosci.* 33 12825–12834. 10.1523/JNEUROSCI.0783-13.2013 23904617 PMC3728690

[B95] Zhang Y. Zhai Q. Luo Y. Dorf M. E. (2002). RANTES-mediated chemokine transcription in astrocytes involves activation and translocation of p90 ribosomal S6 protein kinase (RSK). *J. Biol. Chem.* 277 19042–19048. 10.1074/jbc.M112442200 11893739

[B96] Zhou Y. Won J. Karlsson M. G. Zhou M. Rogerson T. Balaji J. (2009). CREB regulates excitability and the allocation of memory to subsets of neurons in the amygdala. *Nat. Neurosci.* 12 1438–1443. 10.1038/nn.2405 19783993 PMC2783698

[B97] Zhou Z. Okamoto K. Onodera J. Hiragi T. Andoh M. Ikawa M. (2021). Astrocytic cAMP modulates memory via synaptic plasticity. *Proc. Natl. Acad. Sci. U.S.A.* 118:e2016584118. 10.1073/pnas.2016584118 33452135 PMC7826339

[B98] Zhuang Z. Yang B. Theus M. H. Sick J. T. Bethea J. R. Sick T. J. (2010). EphrinBs regulate D-serine synthesis and release in astrocytes. *J. Neurosci.* 30 16015–16024. 10.1523/JNEUROSCI.0481-10.2010 21106840 PMC3073557

